# Chemosensitivity and Endocrine Sensitivity in Clinical Luminal Breast Cancer Patients in the Prospective Neoadjuvant Breast Registry Symphony Trial (NBRST) Predicted by Molecular Subtyping

**DOI:** 10.1245/s10434-016-5600-x

**Published:** 2016-10-21

**Authors:** Pat Whitworth, Peter Beitsch, Angela Mislowsky, James V. Pellicane, Charles Nash, Mary Murray, Laura A. Lee, Carrie L. Dul, Michael Rotkis, Paul Baron, Lisette Stork-Sloots, Femke A. de Snoo, Jennifer Beatty

**Affiliations:** 1 Nashville Breast Center, Nashville, TN USA; 2 Dallas Surgical Group, Dallas, TX USA; 3 Coastal Carolina Breast Center, Murrells Inlet, SC USA; 4 Virginia Breast Center, Bon Secours Cancer Institute, Richmond, VA USA; 5 Northeast Georgia Medical Center, Gainesville, GA USA; 6 Akron General Hospital, Akron, OH USA; 7 Comprehensive Cancer Center, Palm Springs, CA USA; 8 St. John Region, Grosse Pointe Woods, MI USA; 9 Northern Indiana Cancer Research Consortium, South Bend, IN USA; 10 Breast and Melanoma Specialists of Charleston, Charleston, SC USA; 11 Department of Medical Affairs, Agendia Inc, Irvine, CA USA; 12 The Breast Place, Charleston, SC USA

## Abstract

**Purpose:**

Hormone receptor-positive (HR+) tumors have heterogeneous biology and present a challenge for determining optimal treatment. In the Neoadjuvant Breast Registry Symphony Trial (NBRST) patients were classified according to MammaPrint/BluePrint subtyping to provide insight into the response to neoadjuvant endocrine therapy (NET) or neoadjuvant chemotherapy (NCT).

**Objective:**

The purpose of this predefined substudy was to compare MammaPrint/BluePrint with conventional ‘clinical’ immunohistochemistry/fluorescence in situ hybridization (IHC/FISH) subtyping in ‘clinical luminal’ [HR+/human epidermal growth factor receptor 2-negative (HER2−)] breast cancer patients to predict treatment sensitivity.

**Methods:**

NBRST IHC/FISH HR+/HER2− breast cancer patients (*n* = 474) were classified into four molecular subgroups by MammaPrint/BluePrint subtyping: Luminal A, Luminal B, HER2, and Basal type. Pathological complete response (pCR) rates were compared with conventional IHC/FISH subtype.

**Results:**

The overall pCR rate for ‘clinical luminal’ patients to NCT was 11 %; however, 87 of these 474 patients were reclassified as Basal type by BluePrint, with a high pCR rate of 32 %. The MammaPrint index was highly associated with the likelihood of pCR (*p* < 0.001). Fifty-three patients with BluePrint Luminal tumors received NET with an aromatase inhibitor and 36 (68 %) had a clinical response.

**Conclusions:**

With BluePrint subtyping, 18 % of clinical ‘luminal’ patients are classified in a different subgroup, compared with conventional assessment, and these patients have a significantly higher response rate to NCT compared with BluePrint Luminal patients. MammaPrint/BluePrint subtyping can help allocate effective treatment to appropriate patients. In addition, accurate identification of subtype biology is important in the interpretation of neoadjuvant treatment response since lack of pCR in luminal patients does not portend the worse prognosis associated with residual disease in Basal and HER2 subtypes.

The Neoadjuvant Breast Registry Symphony Trial (NBRST) is a prospective, phase IV registry study where neoadjuvant chemotherapy (NCT) and neoadjuvant endocrine therapy (NET) regimen outcomes are evaluated, both as response to treatment at the time of surgery and longer term at 5 years.^1^ Since tumors are classified by gene expression array with the molecular subtyping profile BluePrint as well as the MammaPrint prognostic profile, response to treatment according to conventional clinical versus molecular classification can be compared. Phase IV studies are important because they document outcomes after new technology becomes widely available in clinical practice. Although phase III randomized trials are usually required to make major changes in practice (with some emerging possible exceptions, such as basket trials), phase IV experience often supports refined applications for approved technology and can generate important new hypotheses.

The NBRST has enrolled over 1000 patients at a time when the treatment of patients in the neoadjuvant setting has become standard, not only for large inoperable breast cancer that may become operable by downstaging but also for earlier-stage cancer providing a personalized measure of effectiveness against the actual tumor in the individual patient. The NBRST provided physicians and patients with molecular prognostic information to potentially guide treatment allocation and provide a molecular understanding of response to treatment or lack thereof.

Hormone receptor-positive (HR+)/human epidermal growth factor receptor 2-negative (HER2−) tumors remain a challenge for determining best treatment, especially since a subset has substantial benefit with chemotherapy. The 2015 St. Gallen Expert Consensus considers NET the preferred treatment for Luminal A-type postmenopausal patients.^2^ Endocrine therapy can also have an important role in the neoadjuvant setting where systemic treatment may be indicated for several months prior to surgery in postmenopausal women with large and/or technically inoperable tumors. This treatment is intended to shrink the tumor so that in locally advanced disease surgery becomes possible, and in large operable breast cancers breast-conserving surgery can be performed.^3^,^4^ However, large, prospective, randomized, neoadjuvant trials in HR+ patients with NET are still in progress. Response to treatment is more difficult to define in HR+ breast cancer patients; pathological complete response (pCR) is less likely to occur in the first place, and it is not a surrogate endpoint for survival in these patients.^5^ More importantly, no robust formal definition of meaningful response to NET is available. Quantitative measurements of response rely on indirect assessments. While ‘clinical response’ refers to the decrease in tumor size, ‘pathological response’ can detect a meaningful decrease in tumor cellularity with an increase in fibrosis or formation of fibrous connective tissue. More complications arise from cases where these definitions are discordant in approximately 20 % of tumors.^6^ Physicians still rely on clinical response during treatment in daily practice.

Functional molecular subtyping with the 80-gene BluePrint assay and 70-gene MammaPrint assay was developed to improve biological identification for better treatment assignment (to responsive patients), and further dissection of patient groups wherein additional treatment options should be evaluated in future trials. Identification of a group of patients where NET is effective can avoid unnecessary toxicity when the same group is minimally responsive to chemotherapy. BluePrint subtyping classifies patients into the following subgroups: Luminal, HER2, and Basal type. The group of genes identifying Luminal-type breast cancer is highly enriched for genes having an estrogen receptor (ER) binding site proximal to the promoter region, suggesting that these genes are direct targets of the ER.^7^ MammaPrint combined with BluePrint can substratify luminal subtype patients into Luminal A and Luminal B groups. MammaPrint has recently provided level 1A evidence for identification of patients with low recurrence risk and negligible chemotherapy benefit,^8^ and BluePrint molecular subgroups had distinctly different outcomes in retrospective analyses from four NCT trials. Luminal A patients have a low pCR rate of 6 % to NCT and an excellent distant metastasis survival of 93 %.^9^

The NBRST trial results allow us to determine if physicians and patients incorporate such findings in daily clinical practice, and help answer important practical questions such as how often do physicians choose NET in clinical luminal patients, and is there a difference in clinical characteristics for patients who receive NCT versus NET. The NBRST also documents the molecular subtype for clinical luminal patients, the pCR rate to NCT and the clinical response to NET in these different molecular subtypes, and correlates MammaPrint results in luminal patients with pCR to chemotherapy.

## Patients and Methods

### Patients

Patients with breast cancer from 62 US institutions who had started, or were scheduled to start, NCT or neoadjuvant hormone therapy, after successful MammaPrint/BluePrint assay, were enrolled in the prospective NBRST registry trial between June 2011 and November 2014. Patients with T4 or inflammatory disease were eligible for inclusion. Excluded from the study were patients who had an excisional biopsy or axillary dissection, confirmed distant metastatic disease, any prior chemotherapy, radiotherapy, or endocrine therapy for the treatment of breast cancer and any serious uncontrolled intercurrent infections or other serious uncontrolled comorbid disease. The trial was approved by Institutional Review Boards in all participating centers, and was registered with ClinicalTrials.gov (identifier NCT01479101). Before registration, all patients provided signed informed consent for the trial and for research on their tumor samples. Treatment was at the discretion of the physician adhering to either National Comprehensive Cancer Network (NCCN)-approved regimens or other peer-reviewed established regimens. No specific recommendations were given for the selection to treat patients with neoadjuvant treatment. The NBRST registry is a unique, large database of US patients in a wide variety of clinical practice settings that provides insight into outcomes associated with molecular tumor type and systemic treatment for this neoadjuvant treatment-eligible population. For the current substudy, only locally assessed immunohistochemistry/fluorescence in situ hybridization (IHC/FISH) HR+ , HER2− patients were included.

### Molecular and Clinical Characteristics

The 70-gene expression profile MammaPrint and the 80-gene molecular subtyping profile BluePrint were assessed from the fresh or formalin-fixed core needle biopsy at the centralized Agendia Laboratory blinded for clinical and pathological data. Microarray analysis (RNA labeling, microarray hybridization, and scanning) was performed on the RNA, which was cohybridized with a standard reference to the custom-designed diagnostic chip, each containing oligonucleotide probes for the profiles in triplicate or more.^7^,^10^

Four distinct molecular subgroups —Luminal A type, Luminal B type, HER2 type, and Basal type—were identified and used for further analysis. In this study, we defined Luminal A-type tumors as Luminal type by BluePrint with a low risk score by MammaPrint, and Luminal B-type tumors as BluePrint Luminal type with a MammaPrint high risk score.

HR status (ER and progesterone receptor [PR] status) and HER2 status were determined locally on pretreatment core biopsies. Both ER and PR status were determined by IHC and were considered positive if there was ≥1 % positive staining.

### Objectives and Endpoints

The primary endpoint for patients who received NCT was pCR, which is defined as the absence of invasive carcinoma in both the breast and axilla at microscopic examination of the resection specimen, regardless of the presence of carcinoma in situ (ypT0/isN0). All pCRs were verified with a de-identified copy of the surgical pathology report.

The primary objective for patients who received NET was clinical response rate, which was defined as the proportion of patients who achieved a complete or partial response at any time before surgery.

Tumor assessments at baseline, before surgery, at the final visit, or at withdrawal were carried out by magnetic resonance imaging (MRI), ultrasound, mammography, clinical breast examination (CBE), or other conventional methods as per local practice.

### Statistical Analysis

Baseline characteristics, including age, menopausal status, ER/PR status, T stage, grade, nodal involvement and histology, as well as MammaPrint and BluePrint results were summarized in an incidence table. This exploratory analysis was undertaken for both neoadjuvant treatment groups (NCT and NET). A *χ* ^2^ test was performed for comparison of a categorical variable between both treatment groups, and Fisher’s exact test was used when a cell contained <5. A non-parametric test was used to compare medians of the continuous variables. A significant finding was defined as a *p* value below 0.05.

Univariate logistic regression analyses of pCR to NCT were evaluated to identify individual patient and tumor prognostic factors. Significant factors from the univariate analyses were included in a multivariate modeling procedure. The probability of pCR as a function of the MammaPrint index was calculated. All calculations were performed using IBM SPSS Statistics 22.0 (IBM Corporation, Armonk, NY, USA).

## Results

A total of 474 eligible patients with IHC/FISH HR+/HER2− tumors were enrolled in the NBRST study. MammaPrint classified 29 % of patient samples as low risk and 71 % as high risk, while BluePrint classified 29 % of patient samples as Luminal A type, 53 % as Luminal B type, and 18 % as Basal type.

Overall, 405 patients were treated with NCT, 61 were treated with NET, and 8 received both NCT and NET. Table 1 lists the pretreatment patient and tumor characteristics for the NCT and NET groups.

**Table 1:** Pre-treatment clinical characteristics and treatment regimens (*n* = 466^a^, HR+/HER2−)

	NCT (*n* = 405)	NET (*n* = 61)	*p* value
Median age, years (range)	51 (22–79)	71 (43–88)	**<0.001**
Pre- and perimenopausal^b^	196 (48)	5 (8)	**<0.001**
Postmenopausal^c^	209 (52)	56 (92)	
T1/T2	268 (66)	47 (77)	0.091
T3/T4	137 (34)	14 (23)	
Clinically LN+	254 (63)	16 (26)	**<0.001**
Grade 1/2	197 (49)	51 (84)	**<0.001**
Grade 3	190 (47)	6 (10)	
Grade unknown	18 (4)	4 (7)	
Invasive ductal carcinoma	336 (83)	43 (70)	**0.001**
Invasive lobular carcinoma	46 (11)	17 (28)	
Other	23 (6)	1 (2)	
ER status (IHC)+	388 (96)	61 (100)	0.146
PR status (IHC)+	316(78)	54 (89)	0.063
MammaPrint low risk	95 (23)	20 (33)	
MammaPrint high risk	310 (77)	41 (67)	
BluePrint Luminal A type	95 (23)	41 (67)	
BluePrint Luminal B type	224 (55)	19 (31)	
BluePrint HER2 type	1 (<1)	–	
BluePrint Basal type	85 (21)	1 (2)	
AC-T or TAC	175 (43)	–	
ddAC—T	113 (28)	–	
TC	65 (16)	–	
AC	16 (4)	–	
Other NCT regimen	36 (9)	–	
Anastrozole	–	34 (56)	
Letrozole	–	15 (25)	
Tamoxifen	–	7 (11)	
Exemestane	–	2 (3)	
Other		3 (5)	

Significant values are given in bold at *p* ≤ 0.05

Data are expressed as *n* (%) unless otherwise specified

^a^8 Patients had NCT and NET

^b^Pre- and perimenopausal: 6–12 months since last menstrual period

^c^Postmenopausal: >12 months since last menstrual period or bilateral oophorectomy/hysterectomy

*ER* estrogen receptor, *PR* progesterone receptor, *IHC* immunohistochemistry, *A* doxorubicin, *T* taxane, *C* cyclophosphamide, *HR*+ hormone receptor-positive, *HER2*− human epidermal growth factor receptor 2-negative, *LN* + lymph node-positive, *NCT* neoadjuvant chemotherapy, *NET* neoadjuvant endocrine therapy

Patients in the NCT group were, on average, 20 years younger, and 50 % were premenopausal. NCT patients had more positive lymph nodes (63 vs. 26 %; *p* < 0.001) and had a breast cancer with a higher histological grade (grade 3: 47 % vs. 10 %; *p* < 0.001).

Patients treated with NCT more often had a high-risk profile according to MammaPrint, compared with patients treated with NET (77 vs. 67 %; *p* < 0.001), which resulted in a higher amount/number of patients with Luminal B tumors within the NCT group (55 vs. 31 %). According to BluePrint, one-fifth of the NCT group was Basal type.

### Neoadjuvant Chemotherapy

Review of the chemotherapy regimens showed that the most commonly used regimen was AC-T (doxorubicin/cyclophosphamide followed by a taxane) or TAC (docetaxel/doxorubicin/cyclophosphamide) [43 %], followed by dose dense AC-T (28 %), and TC (docetaxel/cyclophosphamide) [16 %].

Overall, 46 (11 %) patients did not complete all planned NCT cycles. Two patients died during NCT (septicemia and encephalitis infection), 29 stopped early because of toxicities, 7 stopped early because of tumor progression or lack of response, 3 patients and 1 medical oncologist decided to proceed to surgery before completion of all cycles, and no reason was specified for the remaining 4 patients.

**Fig. 1:**
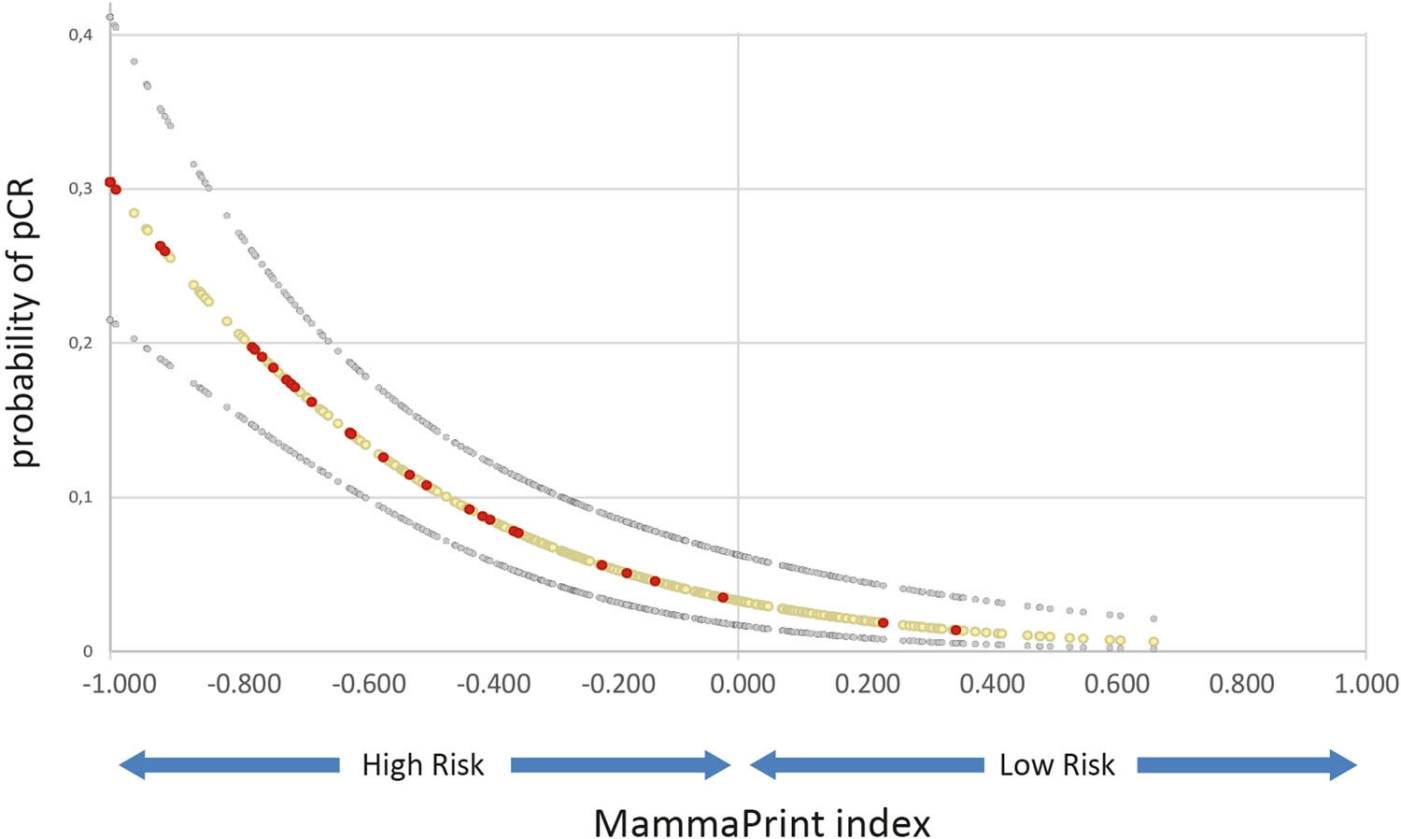
Probability of pCR (ypT0/isN0) to NCT for the MammaPrint index (*n* = 405), and probability of pCR as a function of the MammaPrint index. The *red* and *grey circles* represent patients who did and did not have a pCR, respectively. The MammaPrint index is positively associated with the likelihood of pCR (*p* < 0.001), suggesting that patients who are at the highest risk of recurrence are more likely to have chemotherapy benefit. *pCR* pathological complete response, *NCT* neoadjuvant chemotherapy

The overall pCR (ypT0/isN0) rate to NCT was 11 %. Only 2 of 95 (2 %) patients with a MammaPrint low-risk tumor had a pCR, while significantly more patients with high-risk tumors had a pCR (13 %; *p* = 0.001). Figure 1 shows how the MammaPrint index was highly associated with the likelihood of pCR (*p* < 0.001), suggesting that patients with tumor samples at highest risk of recurrence are more likely to have chemotherapy benefit.

**Fig. 2:**
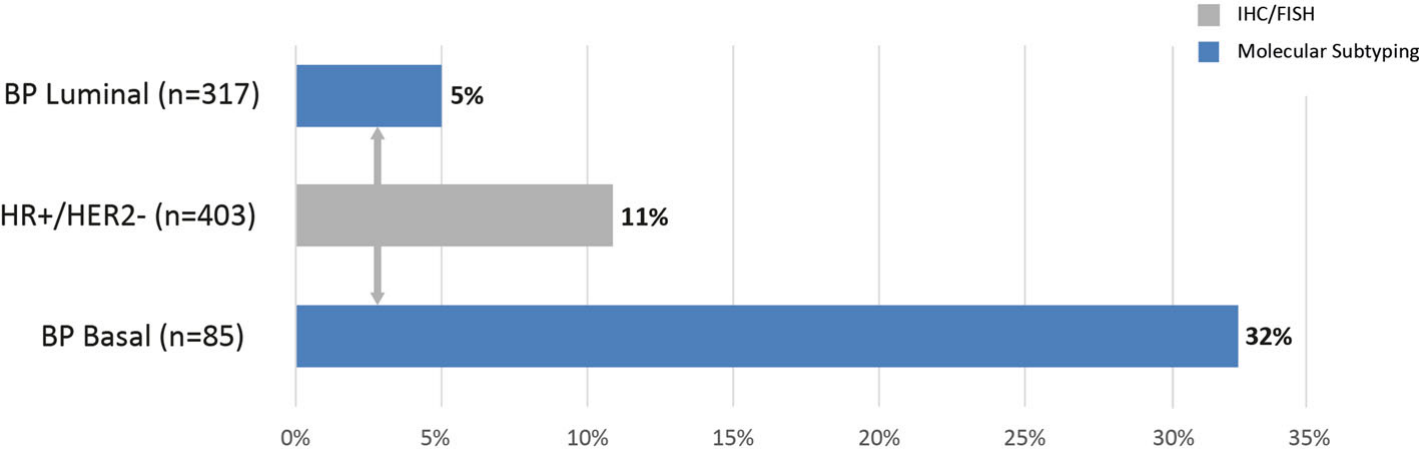
Chemosensitivity (pCR) per subtype classification (*n* = 403). One patient was classified as HER2 type, but this patient did not have a pCR. *pCR* pathological complete response, *HER2* human epidermal growth factor receptor, *BP* BluePrint, *HR*+ hormone receptor-positive, *IHC* immunohistochemistry, *FISH* fluorescence in situ hybridization

The pCR rate for the BluePrint Luminal subtype was only 5 %, and statistically significantly less than the pCR rate of 32 % for the 85 clinical IHC/FISH HR+/HER2− patient samples classified as Basal subtype (*p* < 0.001) (Fig. 2).

The following factors were found to be significantly (*p* < 0.05) associated with the odds of achieving pCR based on univariate logistic regression analyses (see Table 2): tumor grade, PR status, MammaPrint result, and BluePrint result. In addition, the following factors were independently associated with the odds of achieving pCR based on multivariate logistic regression modeling: BluePrint (*p* = 0.005) and grade (*p* = 0.045).

**Table 2:** Univariate and multivariate analysis of patient and tumor characteristics associated with pCR (ypT0/isN0) (*n* = 405)

Characteristic	Univariate OR (95 % CI)	Univariate *p* value	Multivariate OR (95 % CI)	Multivariate *p* value
Age	0.985 (0.959–1.012)	0.276		
Menopausal status	1.396 (0.739–2.638)	0.304		
cT stage	0.486 (0.226–1.045)	0.065		
c Lymph nodes	0.723 (0.381–1.369)	0.319		
Grade	6.353 (2.75–14.675)	**0.000**	2.615 (1.009–6.777)	**0.048**
Histology	0.334 (0.078–1.431)	0.140		
ER	0.891 (0.197–4.037)	0.881		
PR	0.171 (0.088–0.331)	**0.000**	0.479 (0.216–1.063)	0.070
MammaPrint	7.140 (1.694–30.101)	**0.007**	1.922 (0.420–9.438)	0.385
BluePrint-subtype	8.758 (4.440–17.273)	**0.000**	3.301 (1.422–7.666)	**0.005**

Significant values are given in bold at *p* ≤ 0.05

*pCR* pathological complete response, *OR* odds ratio, *CI* confidence interval, *ER* estrogen receptor, *PR* progesterone receptor

### Neoadjuvant Endocrine Therapy

Overall, 34 of 69 NET patients (56 %) received anastrozole as NET, followed by letrozole (*n* = 15, 25 %) and tamoxifen (*n* = 7, 11.5 %) (Table 3).

**Table 3:** Clinical response and duration to the neoadjuvant endocrine therapy regimens

Regimen	*N* (%)	Mean duration (weeks)	Clinical response determined by physician (*n*)			
			CR	PR	SD	PD
Anastrozole (A)	34 (56)	29 (4–83)	1	22	11	–
Letrozole (L)	15 (22)	25 (7–69)	–	10	4	1
Exemestane (E)	2 (3)	46 (35–57)	1	1	–	–
Letrozole → exemestane	2 (3)	27 (17–36)	–	–	1	1^a^
Anastrozole → exemestane	1 (2)	26	–	1	–	–
Tamoxifen	7 (10)	26 (4–74)	–	2	5	–

*CR* complete response, *PR* partial response, *SD* stable disease, *PD* progressive disease

^a^BluePrint Basal-type patient

All but one patient had a BluePrint Luminal tumor. One patient had a BluePrint Basal-type tumor and this patient progressed on letrozole followed by exemestane.

**Fig. 3:**
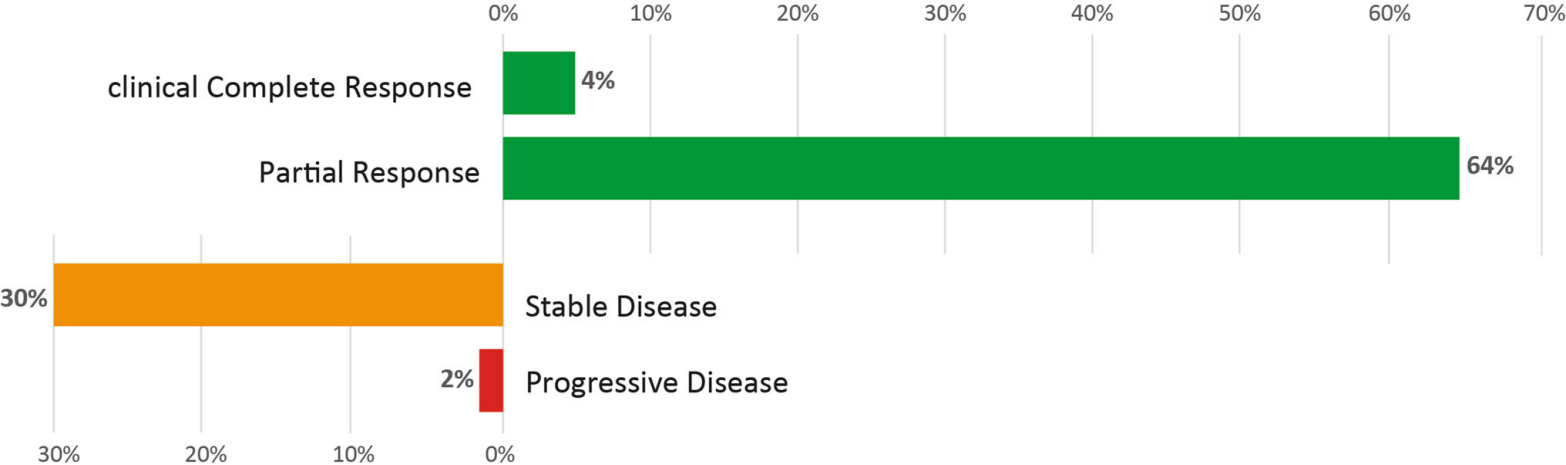
Clinical response rate (cCR and PR) to neoadjuvant endocrine therapy with an aromatase inhibitor in BluePrint Luminal tumors (*n* = 53). *cCR* clinical complete response, *PR* partial response

Fifty-three patients with BluePrint Luminal tumors received NET with an aromatase inhibitor and 36 (68 %) had a clinical response (Fig. 3). Seven patients received tamoxifen as NET and two (29 %) had a clinical response. Patients with Luminal A tumors (MammaPrint Low Risk) had the same clinical response rate (68.6 %; 24/35) to NET as patients with Luminal B (MammaPrint high risk) tumors (66.7 %; 12/18).

### Surgery

Overall, 99 % of enrolled patients underwent surgery; 39 % had a lumpectomy or segmental resection and 61 % had a mastectomy. Patients who were treated with NET had a lumpectomy or segmental resection rate of 52.5 % (32/61), which is significantly higher than the 37 % rate in patients who received NCT (*p* = 0.0098).

## Discussion

In the prospective neoadjuvant NBRST study, 405 (85 %) patients with HR+/HER2− tumors received NCT, and the overall pCR (ypT0/is/N0) rate was 11 %. Only 2 of 95 (2 %) patients with MammaPrint low-risk tumors had a pCR, while significantly more patients with high-risk tumors had a pCR (13 %; *p* = 0.001). The MammaPrint index was highly associated with the likelihood of pCR (*p* < 0.001), suggesting that patients with tumors at the highest risk of recurrence are more likely to have chemotherapy benefit.

BluePrint functional subtyping revealed that 18 % of patients with locally assessed HR+/HER2− tumors were BluePrint Basal type, with a significantly higher response rate of 32 % compared with the 5 % of BluePrint Luminal-type cases. Multivariate logistic regression showed that BluePrint and grade were found to be significantly associated with the odds of achieving pCR. This confirms, in a wide range of practice settings, the approximately 1-in-5 reclassification rate for ‘clinical luminal’ tumors that has been previously described for BluePrint,^1^ as well as by others.^11^ Molecular classification of these tumors indicates a Basal-type make-up despite positive ER staining. These tumors may lack a functional response to estrogen and consequently respond more like triple-negative tumors, therefore benefit from chemotherapy for these patients should be considered likely.

Patients with a true Luminal-type tumor can be good candidates for NET. The current study included 53 patients with BluePrint Luminal tumors who received NET with an aromatase inhibitor. Of these 53 patients, 36 (68 %) had a clinical response. Patients with this tumor type do not demonstrate the correlation between disease-free survival and pCR seen with Basal and HER2 types. In fact, those with Luminal A type have an excellent prognosis in spite of their low pCR rate.^8^ These findings are also in accord with the recently reported prospective, randomized, phase III study MINDACT, which evaluated 6693 women with stage T1–T3 operable breast cancer with 0–3 nodes involved, in which 64 % of women had a MammaPrint low risk of recurrence. These patients (including 48 % with positive nodes) had a 5-year distant metastases-free survival of 95 %, irrespective of the use of adjuvant chemotherapy.^7^

## Conclusion

MammaPrint and BluePrint reclassify 18 % (87/474) of patients compared with conventional assessment (1 HER2-type patient and 86 Basal-type patients). These patients have a significant higher response rate to NCT compared with BluePrint Luminal patients, while BluePrint Luminal patients have an excellent partial response rate to NET.
